# Direct observation in postgraduate training: making it happen and making it work

**DOI:** 10.1111/medu.13755

**Published:** 2018-11-14

**Authors:** Nienke Renting

**Affiliations:** ^1^ Faculty of Economics and Business University of Groningen Groningen the Netherlands

## Abstract

How does one successfully implement direct observation of residents’ competencies in postgraduate training programs? Renting offers insights into how to make it work.

Successful organisation of direct observation of residents by attending physicians may make or break the implementation of competency‐based approaches in postgraduate training programmes. It seems, however, that direct observation of competencies does not readily fit into most postgraduate programmes, as it often happens infrequently and is of poor quality. Direct observation serves three important purposes in competency‐based training. Firstly, direct observation is the foundation of all valid and reliable workplace‐based assessment tools.^1^, ^2^ Secondly, feedback provided after direct observation is one of the most powerful learning tools in residency.^3^ Finally, direct observation helps establish relationships and build mutual trust between residents and attending physicians.^4^ Promoting assessment, feedback and supervisory relationships are three good objectives that justify the quest for increased frequency and improved quality of direct observation.

Promoting assessment, feedback and supervisory relationships are three good objectives that justify the quest for increased frequency and improved quality of direct observation

In this issue, Gauthier et al.^5^ investigate residents’ and attending physicians’ perceptions of direct observation before transitioning to a competency‐based approach in their postgraduate internal medicine training programme. Gauthier et al.^5^ find that many internal medicine residents and attending physicians articulated a quite narrow perspective of direct observation. Direct observation to them typically entails planned encounters when an attending physician sits in and witnesses a resident during direct patient contact, for instance when taking a history or performing a physical examination. By contrast with direct observation, when probed, some of the participants in Gauthier et al.^5^ described ‘informal observation’ as occurring much more frequently and in an ad hoc manner. Informal observation entails, for instance, handovers, managing cases and interactions with other health care professionals. Basically, almost any professional situation that occurs during day‐to‐day collaboration of attending physicians and residents, on the ward and in the outpatient clinic, might be suitable for direct observation if framed more broadly, by including what the participants mentioned as ‘informal observation’. By reframing direct observation more broadly, suddenly competency‐based postgraduate training becomes a lot less time consuming and a lot more feasible.

By reframing direct observation more broadly, suddenly competency‐based postgraduate training becomes a lot less time consuming and a lot more feasible

I warmly embrace the advice of Gauthier et al.^5^ to reframe ‘direct observation’ much more broadly and to incorporate also professional situations outside of patient encounters. In fact, that is exactly what we did when we transitioned our postgraduate internal medicine programme to a competency‐based approach. We developed a feedback system that included direct observation of residents in a variety of professional situations to provide them with immediate and specific feedback.^6^ Five authentic professional situations were determined to together cover all CanMEDS roles. Structuring observation and formative feedback in this way helped to transition towards competency‐based training. The system helped attending physicians to provide high‐quality specific feedback on the defined CanMEDS roles. Furthermore, it ensured attention beyond medical expertise, including roles that had not been part of medical training for a long time. This was a very important finding, given that many programmes still struggle to sufficiently incorporate considered ‘difficult’, CanMEDS roles such as collaborator or leader that cannot directly be observed during patient encounters.

So how can we stimulate more and better direct observation in our training programmes? Direct observation should be approached systematically and achieved with careful consideration of organisational culture and values, in order to be able to tackle inevitable obstructions.^7^ The participants in Gauthier et al.^5^, described reduced efficiency as an important hindrance to the implementation of (more) direct observation. Furthermore, both residents and attending physicians seem to have their reasons for refraining from initiating direct observation. Residents have a significant amount of anxiety during direct observation, a fear of possible consequences if deficiencies are uncovered and a fear of bothering busy attending physicians too much.^5^, ^8^ Simultaneously, attending physicians also hesitate to initiate direct observation because they are afraid residents might feel mistrusted and worry that their autonomy will be jeopardised.^4^ Therefore, a system with a solid foundation of planned direct observations and added tailored direct observations, where needed, seems the only way to go about it. Regularly planned observations become part of ‘business as usual’ and make direct observation less scary. Additional tailored observations should be mutually agreed on by residents and attendings physicians, and optimally support learning by connecting to residents’ individual learning needs.

Therefore, a system with a solid foundation of planned direct observations and added tailored direct, observations, where needed, seems the only way to go about it

Although direct observation has great learning potential, learning does not occur simply because an attending physician observes an activity. So how can we make sure direct observation realises its full learning potential instead of being just a waste of valuable time? It may very well be that the term ‘direct observation’ still brings an image to mind of what anthropologists have been doing for over a century: essentially becoming a metaphorical ‘fly on the wall’ by quietly observing and affecting the situation as little as possible. An attending physician can never become this unnoticed fly and is, fortunately, much more valuable for residents’ learning when taking on an active role. Direct observation can be bidirectional, where the resident and attending physician naturally take turns in acting and observing during patient encounters based on the residents’ abilities.^4^ Attending physicians can ask probing questions during case presentations to disclose residents’ clinical reasoning.^5^ Attending physicians can provide residents with constructive and specific feedback afterwards, to boost residents’ learning.^6^ Moreover, direct observation contributes to quality of care, functioning as a safety net, as attending physicians can step in whenever needed.^5^

So how can we make sure direct observation realises its full learning potential instead of being just a waste of valuable time?

I imagine many of the attending physicians in the study by Gauthier et al.^5^ and elsewhere, would say that a lot of the above is already occurring in their daily practice. Great! The transition towards a competency‐based approach does not have to be disruptive. *So why adopt a competency‐based approach?* Because competency frameworks may help to better respond to the changing environment of postgraduate training. Workplace‐based learning increasingly affords fragmented, brief contact of residents with multiple attending physicians, who must attempt to quickly assess the residents’ learning needs and professional abilities.^9^ Only systematic direct observation can help make these assessments and aid progressively increased autonomy and trust throughout residency. It also has the potential to aid supervisory handovers from one attending physician to the next, something that happens rarely and causes discontinuity in residents’ learning.^10^ Competency frameworks ideally provide focus during direct observation and become part of a shared language to talk about learning trajectories and performance.

Competency frameworks ideally provide focus during direct observation and become part of a shared language to talk about learning trajectories and performance
